# An interpersonal neurobiology perspective on the mind and mental health: personal, public, and planetary well-being

**DOI:** 10.1186/s12991-023-00434-5

**Published:** 2023-02-03

**Authors:** Daniel J. Siegel, Chloe Drulis

**Affiliations:** Mindsight Institute, Santa Monica, CA USA

## Abstract

This article outlines an Interpersonal Neurobiology (IPNB) perspective on the fundamental components that comprise mental health and promote well-being. The central aim of this paper is to answer essential but often overlooked questions related to the field of mental health, such as: What is the mind? What is the basis of well-being? What is the self and how does it develop? We will offer scientific support for the IPNB position that the mind is relational and embodied and that integration is the basis of mental health. It will also describe how the self extends beyond the individual, arising from and inextricably connected to the social, cultural and planetary systems in which we exist. IPNB is not a form of therapy; rather, it is a framework that focuses on deepening our understanding of the mind and human development across the lifespan. Drawing from interdisciplinary principles from a range of fields including physics, mathematics, neuroscience, and psychology, we will provide a practical view of the underlying basis of mental suffering and the scientific mechanisms of change to improve mental well-being. These core principles are building blocks of clinical evaluation and treatment that can be applied across multiple theoretical orientations and client populations. The special emphasis in this article is on the issue of psychache as an underlying cause of suicide and its relationship to personal, public and planetary health.

## Introduction

There are many collaborative contributors to the growing perspective of cross-disciplinary thinking about the mind and mental health whose empirical research, clinical interventions, and community approaches have shaped its formation, the framework of Interpersonal Neurobiology (IPNB). Now with over eighty textbooks in the Norton Professional series on Interpersonal Neurobiology, this framework offers a wide array of disciplines a unique and useful approach to helping the mind grow and thrive. This article will be written in the first person, through the voice of each of its co-authors—and in these direct entries, we will identify this as in the first-person, singular as either Chloe or as Dan^1^ or in the first-person plural, “we.” We invite you, whatever your own background, to consider these ideas and how they might fit—or not—with your professional work and perhaps even personal life. While the *Archives of Psychiatry* is an academic journal and in this setting the first-person terms of me, you, or we are not often used, it seems that a shift in that usual approach is appropriate as we discuss the nature of the mind itself, the source of our experience of self, identity, and belonging. In this paper, we invite you to explore an overview of this framework of IPNB and how it is potentially relevant to the various disciplines of mental health, including psychiatry, psychology, social work, nursing, and masters level therapists focusing on individuals, couples, families, groups, and communities. As we’ll come to see, while IPNB is not a form of therapy, it informs any form of intervention to promote health by offering a framework for understanding the mind and human development across the lifespan.

One of my (Dan’s) teachers was Edwin Shneidman, Ph.D., a leader in the prevention of suicide who had founded the American Association of Suicidology. A fundamental idea of this pioneer in suicidology and thanatology was that the pain in the psyche—what Professor Shneidman called “psychache”—was a way of naming the experience that he viewed as a fundamental cause of suicide and suicidal ideation. Dr. Shneidman and I similarly shared the view that the field of mental health seemed to be inadvertently reducing individuals to diagnostic categories rather than seeing individuals and their relational worlds. In 1978 when I was a medical student with my first exposure to the working drafts of the *Diagnostic and Statistical Manual of Mental Disorders*, the *DSM* that was about to be published in its third edition by the American Psychiatric Association, I heard that the important step of finding a common vocabulary to communicate about mental illness was paramount. Despite this need for consistency in nomenclature and accuracy in formulation, such a classification system unfortunately may often make the mental health clinician vulnerable to missing the unique features of the persons in their care.

But what are these unique features? If the focus was on the mind, what was this “thing” in our lives, this mind, that was actually experiencing such a range of symptoms and signs revealing at a minimum mental suffering, or, at an extreme, a mental dysfunction so pervasive we might classify it as a disorder? Though I ended up dropping out of medical school in 1980 due to a lack of focus on the mind in medicine, I ultimately came back with a term inside of me, “mindsight,” that would remind me that the mind and its expressions of feelings, thoughts, and meaning, was not only real, but really important. Later studies would show that a capacity to sense the inner state of the person in the clinical setting may be a key feature of therapeutic efficacy [20]. Even primary care physicians who take a moment to identify the internal experience of their patients have improved clinical outcomes, such as enhanced patient immune function and recovery from the common cold a day sooner than control groups who were not offered such empathic comments [22]. Why would sensing the mind—having and showing mindsight—be such a potent component of clinical care? And could this capacity to sense the mind’s inner, subjective nature be related to the specific question about suicide, the psychache that is the pain in the psyche? To address these questions, it seemed that having some clear notions of what psyche and mind might actually be, what truly comprised our mental lives, would be a useful place to begin the journey of the field of mental health.

### The mind and psyche of psychiatry, psychology, and psychological well-being

What exactly is meant by the term, “psyche”? The standard dictionary definition [17] , of this term is the soul, the spirit, the intellect, and the mind. Yet the reality of our various disciplines in the broad field of mental health, ones that often use the prefix, “psyche”—as psychiatrists, psychologists, and those in the psychological fields of psychiatric nursing and social work as well as many other mental health professionals who work as psychotherapists—is that we use the terms “mind” and “psyche” without having been offered a clear definition of what these actually mean. When this first became clear and I, Dan, was being given the opportunity as an educator to meet with hundreds and sometimes thousands of mental health practitioners from around the world, I’d ask them two simple questions about their formal training: “Were you ever offered a definition—not descriptions alone—of what the mind is?”; and, “were you ever offered a definition of mental health?” The result of this survey of one hundred thousand professionals in a wide range of disciplines in the field of mental health were remarkably similar around the globe: two to five percent said “yes” to each question. Taken at face value, what this suggests is that over ninety-five percent of mental health professionals have never been told what the mental or the health means of their professional work in the mental health field.

My own training would put me in the “no” category for each question. We do have many descriptions of the mind’s activities, including the processes of emotions, memories, attention, and thought. The closest to a “definition” that is sometimes offered is the saying, “the mind *is* what the brain does.” This perspective was enunciated by Hippocrates over two thousand years ago (see *The Sacred Disease*) and repeated by William James one over one hundred years ago (see *The Principles of Psychology*). For me, Dan, as a young trainee, back in the early 1980s after I had returned to medical school, entered pediatrics, and then began my psychiatry training, the idea that came from medicine that the mind was just the brain seemed to be a limited part of a much larger story. The brain in our head has a lot to do with our mental life and must have something to do with the psychache that Ed Shneidman was speaking about as a core cause of suicide. But what more might be involved in the mind that experienced so much pain that it would choose to end its own existence? What actually is this psyche, what is this mind, that has an “ache” that could lead individuals to end their lives?

In our various professions that are a part of the field of mental health, we have the opportunity to find a common ground, or what E.O. Wilson [32] might call, “consilience” across a range of independent disciplines that come to similar findings. One of the fascinating implications of these exploratory survey findings is that if the field of mental health does not define the “mental” part of its name, how can it say what the “health” is referencing? In this paper, we begin with the important experience of human suffering and the subjective sensation of psychache and its relationship to suicidal behavior. We will then take a step back and ask the broad question, what is this psyche, this mind, that is experiencing an ache, a suffering so severe that the impulse to end one’s life arises? In short, what is the mind and what is a healthy mind?

### A lack of definition of the mind for the field of mental health

Throughout my (Chloe) training in the second and now third decade of this new millennium, mental health was defined as the absence of mental illness. This limited description reinforces the notion that the sole focus of clinicians is to eliminate the symptoms of disorders. While there are undoubtedly benefits to reducing symptoms, this one-dimensional approach leaves many important questions unanswered: What are the core unmet needs underlying the symptoms? Is the elimination of symptoms the full scope of healing and the goal of clinical intervention? What are the natural resources of the mind and how can they be accessed? These questions are difficult to address if the “mind” and “mental health” remain terms without definition, or at most, simply abstract concepts. If we want to be equipped to reduce the symptoms of mental pain, to facilitate growth and cultivate well-being, we need to have a deeper understanding of what we are talking about when we refer to the mind.

When we communicate with one another with language, we use linguistic terms to convey symbols with meaning—with words. We are beginning with the common term, mind. And we will use other terms, such as “self”, “identity” and “belonging” that each reveal fundamental aspects of our mental lives. As we introduce each of these words in this academic paper, we will try to be as direct and foundational as possible in defining what we mean by the term. Rather than making a citation for every statement, a deeper dive into these ideas and their scientific basis along with the hundreds of research papers supporting their specific details can be found in a number of texts by Dan, including [26–28].

Let’s start here with a common word, “individual.” We might agree that there's a skin encasement around many organs and organ systems that form our body, and that this body is an entity—a thing with boundaries and features—that is the homebase for what we are naming as the individual. Much of the focus in research and clinical interventions in the field of mental health in the last one hundred years has focused on the psychological and neurological processes of the individual as the basis for the mental suffering of those with psychiatric disorders. By neurological, we mean the structure and function of the nervous system including its complex neural interconnections in the brain. By psychological, what is meant are the experiences of thoughts, emotions, memories, meaning, beliefs, attitudes, intentions, and the initiation of action. Some might use the common terms, cognitive, emotional, and behavioral—and there are divisions of clinical intervention, for example, that are named for the specific focus they use, such as cognitive-behavioral therapy or CBT. Groups of individuals can also be the setting of clinical intervention, and even individuals in a family or in a relationship. And in each of these approaches, there is a unit of experience that can be demarcated as the individual human body.

If we were then to assume that those psychological experiences—of emotion, thought, and memory, for example—are simply the output of neurological processes in the head, or even more broadly, in the whole body, then we would locate the psyche only inside the individual. We might even concur with the thousands of year view from medicine that mind is what brain does. While this was the commonly held view at the time I, Dan, was in training, when I was learning to be a psychotherapist, and then when I became an attachment researcher studying parent–child relationships, it seemed to me that whatever this “mind” was, it was more than simply brain activity alone. The mind seemed to be fully embodied, not just up in the head. And there was a relational aspect to mind that was even beyond the individual’s skin-encased body.

In researching attachment, the experience of an infant was directly shaped by communication with parents. The experience of something we could call a “sense of self”—a sense of what a center of experience felt like—was both inside that individual and between that person and others around them. This inner and relational experience of self-raised some provocative questions in my own mind as both a researcher and clinician, as well as an educator and new parent. If the experience we name as self was constructed by the mind, could the mind be *both* inside the individual and relational? If self and the mind it emerged from were in these “two places”, did this mean that the individual body was only part of the self? What could be both within and between?

### Seeking consilience: individual, mind and self

In a range of scientific endeavors to explore self-experience, three features are often highlighted: Sensory subjective experience, Perspective or point of view, and Agency or being a center of initiating action (See *IntraConnected*). These can be readily recalled with the acronym, SPA, the SPA of self-experience. Are these SPA features of self-limited to the individual body? Is the subjective sense we have simply reduceable to “what the brain does?” Here is a story that exemplifies these questions.

When I, Dan, was in college, I worked on a suicide prevention phone service, long before I was to meet Professor Shneidman. On that service, I had the feeling—as the person on the receiving end of the phone line speaking with someone who was thinking of killing themselves—that meaning in their lives, the meaning of life and also the meaning of the words I said, made all the difference in whether they would end their life on that phone call or have a sense of hope and possibility and choose, at least for that moment, to stay alive another day. The meaning of how our relationship unfolded in the communication we shared on that call was literally a matter of life and death. What this meant was that how I focused on the inner life of the caller and then connected in a way that was supportive in our communication would directly influence the inner mental experience of that person. It mattered how we connected in a way that would shape meaning in the individual’s life. All of this—the inner meaning and the meaning of interpersonal connection—seemed far more than what was happening in the brain alone.

So “meaning” is something that seemed to me, as a college student, to really “matter”. In English we have the interesting double meaning of “matter” in that it *matters* and indicates that something is significant; and that matter has substance as something you could hold in your hand and is a real situation, such as a “matter of life and death.” So, we have the intriguing meanings of something that has weight to it, it has significance and substance—it is “substantial”. The overlap of a life that matters, one with meaning and significance, and the lack of these elements of life in suicidal ideation and action, and perhaps found in impediments to mental health in general, can relate to the individual and their place both in life and in the setting of the culture in which the individual lives. This *relational context*—how a person is situated in their connections with other individuals—seems more than just their “setting” but rather an essential feature that matters in their experience of self, their identity, and their sense of belonging. Self we’ve defined with its SPA features of subjectivity, perspective, and agency. Identity can be viewed as those characteristics used to define the self, to identify who we are. Belonging can be described as the experience of being a part of something, to be accepted for one’s unique features while also being a member of something larger than the individual alone. Self, identity, and belonging seem to be three aspects of our mental lives beyond the individual body.

It is these relational factors that seemed to make the experience of being a self, with the subjective sense of being alive, the perspective on life and the world, and the experience of agency with its empowerment and integrity, that were essential for the mental well-being of the individual. When the self is fractured, the psyche aches. Our modern culture, especially prominent in the United States with its extreme emphasis on individuality [18], focuses on the separateness of people. And in this cultural setting, levels of suicide, and the despair of psychache, are high. Following the viral COVID-19 pandemic, suicide levels, along with the incidence of anxiety, depression, loneliness, and addiction, have risen dramatically—especially in youth [7]. But if these are each an example of impediments to mental health, if these are examples of mental suffering, what actually is this mind that is not healthy, this mind that is aching? Is this solely something arising inside the body of the individual because of its isolation? Or, might the self that is suffering be embedded in a much larger system than that of the body alone?

From a cognitive science vantage point, four E’s are used to describe our mental experience of information processing: Embodied, Enacted, Extended (beyond the body), and Embedded (in our social worlds) [19]. And from this perspective, we do not limit information processing to the individual alone. Even your experience of reading this paper, and our experience of writing it, are examples of extended information processing. We share information with one another as individuals.

After finishing my clinical training in adult and then child and adolescent psychiatry, I, Dan, did a research training fellowship through the National Institute of Mental Health studying parent–child relationships and how these interpersonal connections shaped emotions, memory, and narrative. The central role of emotions in our mental life, the ways our past experiences continue to impact us in the present and shape our future within memory processes, and how we find meaning in life through the narrative efforts to make sense of experience each reveal how relationships shape our minds. This was just before and then at the beginning of the Decade of the Brain, as our ability to peer beneath the skull into the neural networks and mechanisms associated with our mental lives was exponentially expanding. Turning to brain studies was now possible, for example, to explore how trauma might specifically impact the encoding, storage, and retrieval of memory in both its implicit and explicit forms. We could even examine the neural networks in the cortical regions involved in the processing of autobiographical narratives—the stories we tell about what we have experienced and who we are. There was clearly a direct association between neural and mental processes as we had known for thousands of years only now the details were becoming possible to envision in useful ways. But if emotions, memory, and narrative are examples of what we experience as “mind”—what actually was this process so fundamental to our well-being? Was the term, mind, simply a placeholder for “brain activity?” And if so, might we simply drop the term, mind, and speak of neural activity instead? Would you mind if we did that and speak, instead, of “would you brain if we did that?” Even if mind was dependent upon that embodied neural activity, at least in part, could it be that what we mean by mind is not the same as the brain?

After that fellowship, the opportunity to run the training program at UCLA in child and adolescent psychiatry also afforded the chance, as the Decade of the Brain, the 1990’s, was unfolding, to invite forty scientists to gather together to address the simple question: “what is the relationship between mind and brain?” The brain was straightforward for the group to define—a complex neural network up in the head, connecting to the whole body. But when it came time to define the mind, short of saying that it is “brain activity,” there were no definitions, only descriptions offered by the academicians. For an anthropologist or sociologist in the room, mind was a social process. For the psychologist or neuroscientist at the meeting, mind was an internal process of the nervous system—the brain up in the head and its neural network activity. The tension in the room was uncomfortable and intense; there seemed little reason to meet again after that first discordant gathering. But after being urged to try one more time, the individuals in the group chose to come again. What would you offer as a definition of the mind that might be a common ground for those who saw mind as social and for those who saw it as neural?

In brief, the challenge of the “what” that could be *both* inside the individual and between the individual and the surrounding world, in relationships with people and with nature, was to imagine what the “stuff” might be that could serve as the basis of mind. What is it that could be both inner and inter? Identifying the essence of neural firing, one finds *energy flow* within neural networks in the form of electrical energy as ions flow in and out of membranes and of chemical energy in the form of neurotransmitters and receptors. Identifying the essence of relationships, one finds the sharing of energy and information flow between individuals and between an individual and the environment. It seemed clear that one way of viewing the common ground of the *neural* view and *relational* view of mind was energy flow. Inner and inter reveal the spatial location of what might be the common “stuff” of the mind: energy flow.

If you see or hear the word, “hello,” this is the energy of light or of sound waves in a specific pattern with symbolic meaning—it is a formation of energy that is “information.” And so, we can say that energy can be in a pure form—like a sunset’s rainbow of color—and we can say “sunset” and we then have a symbolic term, a word, that is representing, it is “re-presenting” or presenting again, the pure form of energy. Some physicists see the fundamental aspect of the universe as energy; others view the universe as comprised of information. To find that common ground, the consilience, we can simply use the term, “energy and information” to be inclusive. In our everyday experience, though, we may more directly sense that information emerges from energy. Because these change, we call this unfolding, “flow.” Energy and information flow, we are suggesting, is the fundamental stuff of the mind—and this flow can be both inner and inter.

### A systems view

In the 1980’s, systems studies revealed that the mathematics of complex systems identified a process known as “emergence” in which the interaction of elements of a system gives rise to something larger than the elements themselves. Could it be that “mind” was an emergent process arising from energy flow that was *both* within and between? In this line of reasoning, neither skull nor skin were impermeable barriers that impeded the flow of energy and information.

One particular emergent process is known as “self-organization.” A system that is capable of being chaotic, open to influences from outside of “itself”, and non-linear, meaning a small input at one time can lead to large and difficult to predict outcomes, is considered a “complex system.” These are systems that adapt and learn, their component elements have interdependent, multidirectional influences on each other, and they have the emergent property of regulating their own unfolding called self-organization. While this may seem counterintuitive in that there is no orchestral director, no guide, no planner of the system’s flow, self-organization governs how a complex system emerges over time. By balancing the differentiation of elements and their linkage, self-organization is optimized, and the system achieves a flowing state toward “maximal complexity.” Within mathematics and the study of probability, one can explore the science of systems and discover how this linkage of differentiated parts is the most probable to arise if nothing impedes its innate emergence. It has been helpful to name this balance of parts being different and parts being linked without losing their differences with a commonplace term, “integration.” Integration is how a complex system achieves optimal functioning—it adapts and learns by way of the intricate interdependent connections among differentiated parts.

While coming from the field of mathematics, systems science can be applied to molecules, bodily systems including the brain, families, communities, and our global ecological biosphere. For me, Dan, this mathematical perspective on systems seemed like it might be both relevant and important in attempting to understand the mind.

The complex system emergent property of self-organization can be imagined visually like a river in which the central flow of integration has the features of being flexible, adaptive, coherent, energized, and stable [26]. In English, this forms a useful acronym, FACES. This FACES set of features describes the harmony of an integrative flow. The two banks outside of this central integrative flow of harmony are chaos on one side, rigidity on the other.
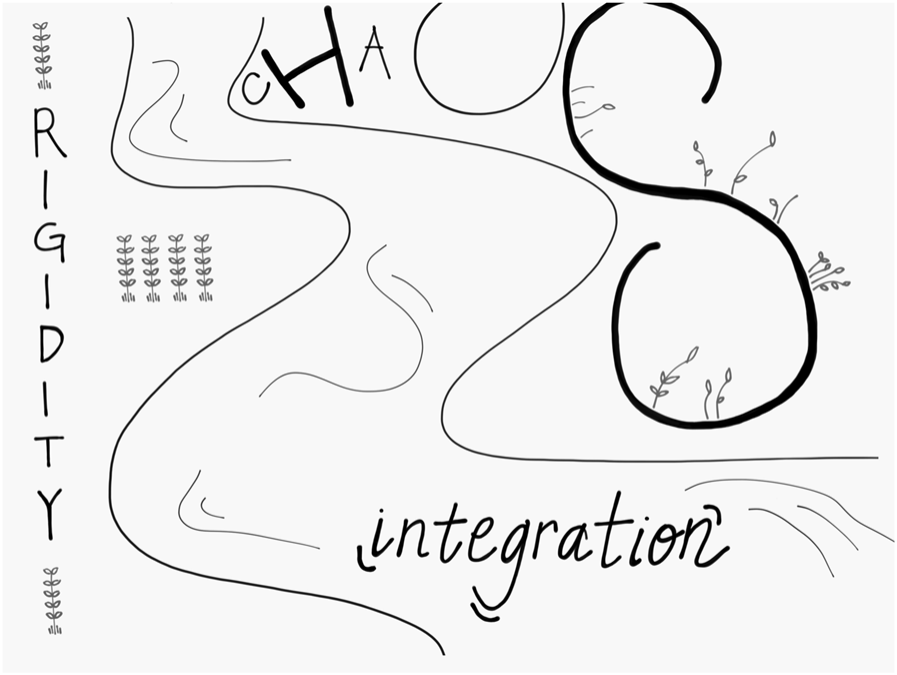


The observation that each of the various mental disorders and its list of symptoms in the ICD-10 or the DSM-V might be re-envisioned as examples of chaos, rigidity, or both, suggested that perhaps the mind, in part, might be defined as: “The emergent, self-organizing, embodied and relational process that regulates the inner and inter flow of energy and information”.

A mind that facilitates integration would be a healthy mind; one that blocks integration by impeding differentiation, linkage, or both, would be an example of an unhealthy mind—or at least a mental state that was leading to mental suffering in the form of chaos and rigidity. The FACES flow and the linkage of differentiated parts that create it results in the experience of harmony. It is this view that leads to the suggestion that integration creates the harmony of health.

The mind also includes other features that may be emergent properties of embodied and relational energy flow. These include subjective experience, the felt texture of life; consciousness, our capacity to be aware; and information processing, how we shape energy flow into symbolic meaning:
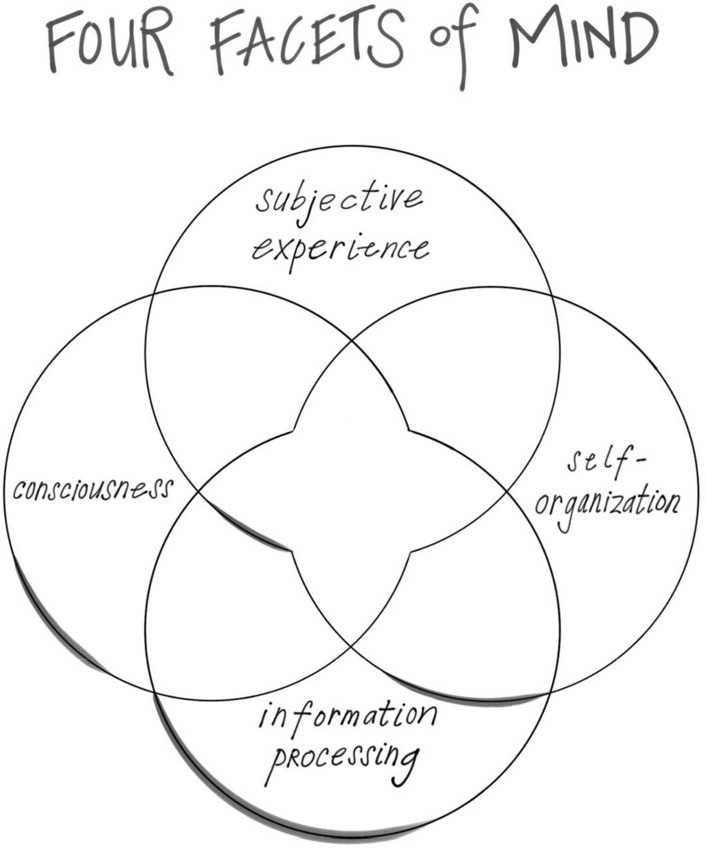


These four facets of mind—subjective experience, consciousness, information processing, and self-organization—enable us to see what an ache in the mind might involve and why it might lead to suicidal behavior and despair. *Energy and information flow that is not integrative leads to chaos and rigidity*. These we can now conceptualize as the fundamental aspects of mental suffering, including that of psychache. How do these arise? From this broad proposal, mental suffering arises from impediments to integration. This blockage of mental health would be revealed as chaos, rigidity, or both. When we are not experiencing integration’s balance in differentiation and linkage, then chaos and rigidity emerge—we can become stuck on the banks of suffering outside the central flow of integration, the harmony of mental health and resilience.

### The mind and psychache

Imagine a situation in which someone calls in to a suicide prevention service and says, “I see no future, I'm in so much pain, I have no feeling of connection to anything, I don't belong anywhere—I see no reason to live.” Now imagine if you are the responder for the service. What would you say? Imagine if, as a trained mental health professional versed in ideas about neurotransmitters and depression, you were to say to them, “I can sense that your serotonin levels are so low in your brain, likely in your prefrontal cortex and your anterior cingulate, that you have a chemical imbalance.” If you were to say only that, even if it were accurate, what do you think might happen? Is this statement connecting with the felt sense, what is sometimes called the “subjective” or “first-person” experience of this distressed individual? Even if it were describing the neural processes underlying those subjective sensations of despair, would the person “feel felt,” a phrase one of my, Dan’s, first patients used to describe what she felt was the attuned connection with her therapist that was the healing component of therapy?

You may sense that this neurochemical statement by itself would be missing an opportunity to attune to the individual human being on the other end of that phone line. Such attunement, such focus of attention on the inner life of another person—or of ourselves for *internal attunement*—would have given them a feeling of connection and hope, they would not feel alone or that they had nowhere or no one with whom they had the feeling of belonging, and they would be less likely to end their lives. And so even if the brain state involved low serotonin levels, naming this fact is not the same as tuning in with your attention to the subjectively sensed experience of *mental* pain. At a minimum, one reason we cannot replace the term “mind” with the terms “brain” or “brain activity” is that our subjective sense of being alive is simply not the same as the electrochemical energy flow of the nervous system.

When we use the term, “subjective”, we in no way view this as less than something that is “objective”—it simply is the inner felt sense of being alive that can only be known directly by the “subject”—the individual feeling the experience of being alive. *Subjective experience* is one facet of mind, and one component of the self. In this way, the subjective sense, along with one’s experience of perspective and agency, form what we’ve seen can be named as the *SPA* of self-experience. The term, “sense of self” is sometimes used to denote this feeling that there is a coherence, a wholeness, to being alive. It is this sense of self that is troubled in the experience of psychache. Though neurochemical status may shape each of the features of our minds, including the SPA of our individual sense of self, they are not the same as how we come to feel whole as a self, how our sensation, perspective, and agency give us a sense of belonging and well-being. In other words, the individual sense of “me” is not the whole story of who we are—who we are is also a relational “we.” In spatial terms, the *me* is inner; and the *we* is inter. Inner and inter are each an aspect of the system of energy flow we are saying is the system of mind.

A second facet of mind, *consciousness*, is how we know we have that pain. This is how we are aware of our inner sense of self. Whatever neural processes underlie the flow of sensation, that create our state of mind, and that shape our experience of being aware—the knowing and the knowns of consciousness are not the *same* as these neural processes. In this fundamental way, our subjective experience and the consciousness that enables us to know that this sensation of being alive cannot be reduced to just brain firing, even if they are internally dependent on them. In other words, mind processes are not the same as brain processes, even if they are dependent upon them, wholly or even in part, for their emergence in our mental lives.

### Emergence, energy, and information

Emergence is something we experience every day, and in the science of complex systems, we see that the arising of something from the interaction of its components that is larger than these fundamental parts is an inherent feature of how these types of systems work. A complex system, one defined as having those three features of being open, capable of chaos, and non-linear, has emergence with which something greater than the individual parts is arising. In IPNB, we propose that the mind in all its facets may be *emergent processes* of embodied and relational energy flow. What this means is that both internal processes, such as our state of physiology and neural firing, as well as communication within our relationships, such as your experience of reading this paper, involve energy flow. Energy is not meta-physical or some non-scientific concept; energy is a core foundation of the physical world. When energy flow has symbolic value, when the energy formation stands for something other than itself, we’ve seen that we call that formation of energy, “information.”

This brings us naturally to our third facet of mind, *information processing*, which may also be an emergent property of energy flow that's happening in a complex system that includes, but may not be limited to, the brain up in our head. When the formation of energy flow symbolizes something, when it “re-presents” something other than itself that is information. Information processing reveals how even a representation is itself a verb-like unfolding—information naturally involves the emergence of even more information as it is “processed” by the emergence of ever more complex representational processes. A breeze on your cheek, can be considered pure energy flow as it exists before we name it, “breeze.” But once you name it the breeze on my cheek, then it becomes information. And once we have that information, other aspects of representations associated with breeze, from ongoing experience, predictions for the future, or memory from the past, will be like a river flowing—that is information processing.

We can get as close to pure energy flow in these bodies we live in with *sensation*. That can be called a “bottom-up” experience in that we have a beginner’s mind, as best we can, to simply experience here-and-now sensory flow of energy, initiated by external or internal sensory processes. Once we take the next step of perceiving something, we are filtering what we perceive by what we’ve learned in the past—a kind of “top-down” filter that shapes our experience of reality, how we construct our view of the world. *Perception* is a top-down construction of information processing.

If we simply stay with these first three facets of mind, there’s nothing about them that helps us define what a healthy mind might actually be. These are useful ways of proposing that energy flow has an emergence to it and that this property of complex systems may be how these mental processes of subjectivity, consciousness, and information processing arise, how they emerge. But what would *healthy* subjectivity, or consciousness, or information processing be?

### Mind as a self-organizing emergent process

When we look into the mathematics of complex systems, we find that these have an emergent property known as “self-organization.” This is a somewhat counter-intuitive way in which assemblies of components that are open, chaos-capable, and non-linear—that make up complex systems—have an innate emergent process that regulates its own becoming as it unfolds over time. Self-organization shapes the state of a complex system, moment-by-moment. By observing that many symptoms of mental disorders could be re-conceptualized as chaos or rigidity, the possibility that the mathematics of complex systems might offer helpful insights into the nature of the mind and mental health became apparent. Mathematics offered a potential way to make sense of the chaos and rigidity that seemed to characterize mental suffering.

This lead to the fourth facet of mind being envisioned and then defined this way:

“An emergent, self-organizing, embodied and relational process that regulates the flow of energy and information.”

Offering this working definition of a facet of the mind to that forty-member group back in the Decade of the Brain enabled us to find common ground and then to go on to meet for four and half years exploring the connections between mind and brain. It was within the collaboration of that multidisciplinary group that the notion of Interpersonal Neurobiology as a framework for understanding the mind and mental health was born.

This definition was useful in helping find consilience across many disparate ways of knowing by locating mind as an emergent embodied *and* relational process. This aspect of this proposal for a definition of the mind means that our mental lives emerge from beyond simply the brain in the head and involve the whole of the body; and mind also emerges within our relationships with people and the whole of the planet. This is a systems view of mind. A unanimous vote supporting this working definition arose from the academic group, enabling a diverse set of forty scientists to find a common ground of which they were previously unaware. Energy flow is not limited by skull nor skin, and so the mental lives we lead could be seen to involve cultural processes in our societies, communication patterns in our families, and physiological and neural processes in our bodies.

The importance of a systems view of mind when it comes to growth and change in therapy was exemplified when I, Chloe, began working with kids who had neurodevelopmental differences. I found it was paramount to understand their brain structure and functioning, as the neurological underpinnings of any given challenge with emotional regulation, sensory processing, motor planning or social communication would inform how to support the needs of each individual child. Central to positive therapeutic change was activating neuroplastic processes in the brain to stimulate learning and growth. But how exactly do such changes in the brain occur? In my experience, the pathway to integrative transformation in the brain was always facilitated through the embodied and relational facets of the mind. This happened in the context of shared experiences via an attuned and trusting connection, where I could sense the inner experience of the child and they could sense my being with them, moment to moment, as we navigated problem solving in the physical and social environment together. Any attempt to singularly control the process rather than join in its unfolding invariably blocked progress, so the therapeutic process required an openness and flexibility to the flow of energy and information between us.

Understanding the mind as a self-organizing emergent process allows us to ask a simple question: how does self-organization become optimized? Mathematics has an answer. When components of complex systems differentiate and then link, optimal self-organization emerges. A key to understanding this process is that in the linkage—as facets of the system interconnect—their differentiation is not lost. In this way, optimal self-organization does not arise with blending or making the system homogenous; instead, there is a balance of differentiation and linkage. In mathematics this combination of differentiation and linkage has no term, and so in IPNB it became important to have a way of naming this process, and the common-language word we use is *integration*.

### Integration as health

Integration, which in IPNB we define as a combination of the linkage and differentiation of parts or facets of a system, is how mathematics views the way a complex system optimizes its self-organization. In other fields, this term may be used for linkage alone (as in neuroscience using “segregation” for differentiation and “integration” for linkage) or as addition in which differentiation is lost with the joining function (as in calculus). From the mathematics of complex systems, optimal self-organization is how the system becomes *flexible*, *adaptive*, *coherent*, which means resilient over time, *energized* and *stable*, meaning it's reliable, not that it's rigid. This combination of characteristics can be remembered with the acronym, FACES, which describes the quality of harmony. Unusual to imagine, but might the mathematical principles of complex systems be applied to understanding the mind? If so, then could mental health—and possibly health in general—be arising with the optimal self-organization that comes from integration?

As described above, this FACES flow of harmony can be visualized as the flow of a river, the central flow as that FACES emergence of being flexible, adaptive, coherent, energized, stable. Outside of this integrative harmony on one bank is *chaos*, and the other bank is *rigidity*.

If one examines the *Diagnostic and Statistical Manual*, for example, it’s possible to reframe the symptoms of each syndrome as either chaos or rigidity. The proposal back from 1992 and published in *The Developing Mind* in 1999 was that perhaps mental un-health—what is called “mental disorder”—is an example of impaired integration in which there is a blocking of differentiation, or linkage, or both. The outcome of such impediments to integration is chaos, rigidity or both. And since that time, each study of individuals with major mental disorders reveals neural structure and function of the brain with impaired integration [26]. For example, individuals with a range of conditions, such as those with schizophrenia, manic-depressive illness, autism spectrum disorder, and post-traumatic stress disorder have impaired functional and structural integration as revealed especially in the integrative regions of the prefrontal cortex, hippocampus, corpus callosum, and the interconnections of the connectome [14, 23, 30]. Of note is that regardless of etiology, whether primarily experiential or what might be considered “innate” or not caused by experience, the finding remains that mental suffering associated with a psychiatric condition appears to have impediments to neural integration, and even to be associated with challenges to relational integration as well. Relational integration emerges as individuals are honored for their differences and connections are established with respectful, compassionate communication.

A study by [29], revealed the corollary, that the most robust predictor of well-being across a wide array of measures is how interconnected the connectome is. The “connectome” is a term for the ways the many differentiated regions of the brain are linked, and so a shorthand for the term “degree of interconnectedness of the connectome” is, more simply, how integrated the brain is. Other studies have also shown that certain ways of training the mind can also lead to the neuroplastic changes associated with well-being—and they make the brain’s differentiated areas more linked. This reveals how what we do with the mind can integrate the structure and function of the brain. What are these practices? When the mind’s *attention* is trained to be focused, when its capacity to open *awareness* is strengthened, and when the mind’s *intention* is set in a direction of kindness, these positive changes emerge. This can be called “three-pillar mind training” [31] and include practices that teach an individual to focus attention, open awareness and build kind intention.

### Mental suffering as chaos or rigidity

If someone is experiencing chaos and rigidity, we can see this as the underlying mechanism of the mental suffering of psychache. This perspective from the consilient view of IPNB enables us to offer a definition of both the mind and of mental health that can help mental health practitioners from our wide range of disciplines to have a common understanding of what we mean by mental suffering and psychiatric dysfunction. This view of mind as both embodied and relational enables us to see that there is no need for a battle between neural views and relational views—each “location of mind” may contribute to the psychache dominated by chaos and rigidity. Chaos can be experienced as intrusive thoughts, waves of intense and dysregulated emotions beyond a “window of tolerance” [26] in which integrative flow is possible, and distressing memories. Rigidity can emerge as a shutting down of an emotional sense of vitality, unrelenting ruminations, compulsive and repetitive behaviors, and disconnection from other people and the accompanying experience of not belonging.

One of the pathways to intervention, whether it is in the acute setting of a suicide prevention service, a psychiatric emergency room, or an outpatient office, is to provide a sense of *connection* by the mental health practitioner’s PART: Their presence, attunement, resonance, and the trust that ensues from these being a part of the communication. In its essence, a therapeutic intervention and connection combine the way each individual in the therapeutic relationship is differentiated from the other and while also becoming linked within compassionate communication. Presence is the open awareness that engenders a receptive relational state, what Steven Porges calls the “social engagement system” [21]. Attunement we’ve seen is the focusing of attention on the inner subjective experience, not merely on the externally visible behaviors. Resonance is feeling the other person’s feelings, without identifying their feelings as your own. And trust is that open state of feeling that the kind intentions and the availability of the other person are reliably present. When we play the PART we need to be *integrative clinicians*, we enable the relational connection to stimulate the internal integration needed for optimal inner regulation.

The benefit of clearly defined language for principles of the mind and the therapeutic alliance has important practical implications. As a play therapy trainee, I, Chloe, needed to explain to parents and teachers how the process of play and emotional attunement were essential for facilitating growth in the embodied and relational mind. These caring adults were accustomed to structured interventions with tangible methods and metrics, as opposed to the emergent, relational process of play therapy. Like many other forms of integrative therapy, much of the work was happening on levels of processing that were not immediately evident to an outside observer. As such, some parents worried this approach seemed trivial or too good to be true to address the complex social, academic, and emotional challenges that their children faced. To gain their trust and collaboration, it was essential that I had language to explain how this process worked and the central role of attunement and presence in cultivating integration.

But being present is not always easy when we face the chaos and rigidity of the minds of others. Keeping integration in mind as not the excessive over-identification with another’s mental suffering but rather the integration that requires both linkage and the necessary differentiation is a helpful starting place. If we over-identify, we lose differentiation; if we overly distance ourselves, we lose connection. Therapy is a fine art of integration in which both differentiation and linkage are highlighted. Having a regular practice that trains the mind to retain this integrative capacity to balance differentiation and linkage is an important resource tool for anyone working on the frontlines of clinical care. Studies reveal [31] that when individuals build the three pillars of focused attention, open awareness, and kind intention, they are able to reduce stress, improve immune function, enhance cardiovascular function, and even reduce bodily inflammation by altering the epigenetic molecules, the non-DNA molecules that sit on top of the genes that regulate the inflammatory response. What one does to train one’s mind in these ways can also optimize the levels of an enzyme called telomerase that repairs and maintains the ends of the chromosomes. And so, with each of these, and especially the latter, molecular mechanisms of well-being, what we do with our mind actually slows the aging process [2]. And, amazingly, three-pillar mind practice has also been shown to enable the structure and function of the brain to become more integrative in the areas we’ve discussed earlier: the corpus callosum that links the differentiated left and right side of the brain, the prefrontal cortex that links the lower areas in the body with the subcortical areas that are often called the brainstem and the limbic areas with the cortex and even with the social world; the hippocampus that grows connections across various differentiated memory systems; and the connectome becomes more interconnected. Yes, an integrated brain corresponds to our experience of well-being. It may not be a surprise then to learn of the important studies on mindfulness training, that include the first two if not all three of these pillars, as a source of preventing burnout in primary care physicians [15]. So why not include this as the basic resource tool for every clinician?

### Integrating consciousness

There is a multi-layered practice that includes all three of these pillars in it. It's called the Wheel of Awareness, and it is possible to learn this and dive deeply into its personal and professional applications [25, 27]. This practice “integrates consciousness” by differentiating its basic components and then linking them to one another. The metaphor of a wheel is useful for imagining the knowns of consciousness on the rim and the knowing of being aware in the wheel’s central hub. A singular metaphoric spoke of attention can then, in imagination, be moved from point to point on the rim. In an advanced step, the spoke is bent back to aim attention directly into awareness itself to invite the experience of being aware of awareness, or simply resting in open awareness without an object of attention.

While this is one example of a practice that includes all three pillars—focusing attention, opening awareness, and building kind intention—the Wheel of Awareness is also an experiential immersion in what a receptive state of mind feels like, and is thus a direct invitation for cultivating an open state of mind we can simply call presence. Research across modalities of psychotherapy suggest that the availability of the clinician to be in an open and receptive state, to be empathic with the patient and ask for and respond non-defensively to feedback, is the best predictor of therapeutic outcome [20]. Finding a practice that suits the individual clinician and then weaving that into a consistent state created during the session can become a trait of the individual which can be very empowering [11]. One of those traits is the capacity to stay present in the face of challenges—one way of defining what it means to be resilient and to have equanimity.

The power of clinicians training their own minds to become more mindful is that it provides a source of inner well-being and resilience as well as the capacity for greater compassion and reduced burnout in the face of connecting with those under their care who are experiencing medical and mental challenges [10].

### The synergy of science, subjectivity, spirituality and service

One of the notable sources of data has been to offer the Wheel of Awareness training to over fifty thousand individuals in person before the viral pandemic of COVID-19. Surveying the responses of the participants and comparing them across the demographics of age, gender, educational background, meditation experience, profession, culture, and nationality, it became evident that there was a common set of experiences individuals would have no matter their background. For example, in responding to an inquiry about the direct first-person experience “in the hub” of the Wheel, common terms offered were: “open, connected to everyone and everything, timeless, empty-but-full, God, eternity, joy, spacious, home, peace.” In taking this first-person reflective data, it becomes “second-person” data by accumulating others’ direct experiences. Then it becomes possible to ask, “how does the subjective experience of many individuals (first- to second-person data) possibly fit with third-person perspectives from empirical science?” In the terms of consilience, is there a common ground that these immersions in the Wheel of Awareness, or other reflective practices, might share with research perspectives?

For example, what might correlate with the symbolic terms, “empty-but-full” or “timeless” or “connected with everything”? In brain science, we might look for neural states that let the skin-barrier not be a defining feature of “self” arise, and these might include a diminishment in the Default Mode Network’s activation thought to be associated with our narrative sense of self across time and of the somatosensory cortex mapping out our bodily sense of self in the present moment [8]. But what might correlate with the timeless sense or the feeling of something being “empty-but-full”?

If the mind is truly an emergent property of energy flow, then turning to the science of energy might be a valid and useful consilient effort to find common ground. In a number of texts (see in particular *IntraConnected*, *Aware*, *Mind*, and *The Developing Mind*, *Third Edition*), there is an in-depth, detailed accounting of this effort to correlate first-, second-, and third-person data into a consilient perspective. For the setting of this presentation, we will simply summarize the major points that help connect subjectivity, science, service and even spirituality into a synergetic whole—a sense of the nature of mind and reality that is larger than any one of these experiences by themselves: There is a synergy to synthesizing them side-by-side.

Here are a dozen Interpersonal Neurobiology Principles that highlight this synergy:

#### The MIND is emergent phenomena of ENERGY flow

The four facets of mind may each be aspects of a complex systems’ emergence—something that arises from the interaction of the components of the system. In the case of the mind, the system is energy flow, and the facets of subjectivity, consciousness, information processing, and self-organization may arise from that flow.
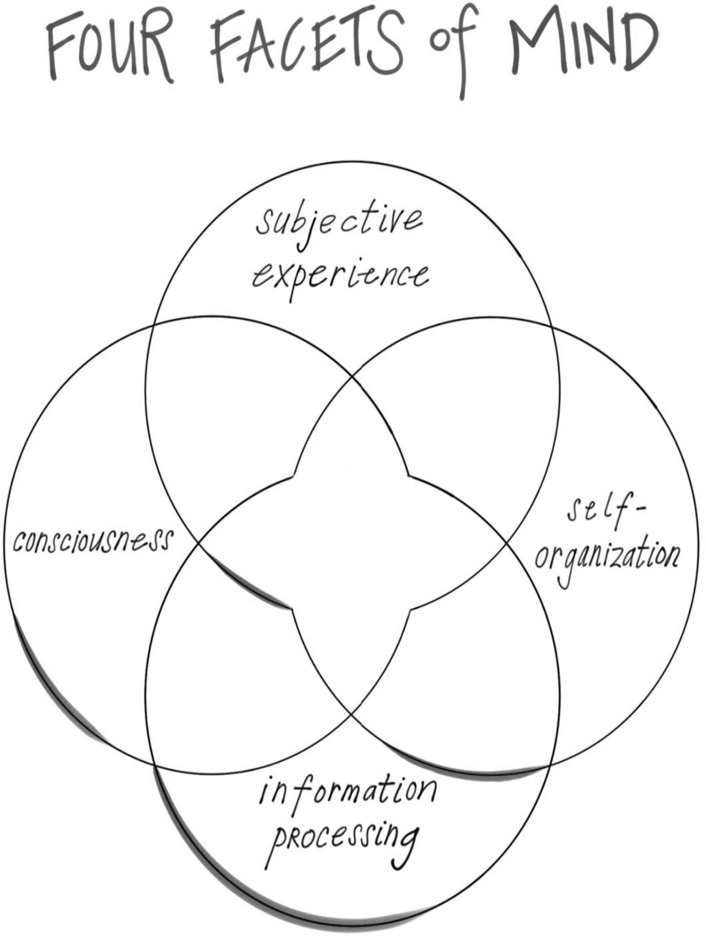


#### Mind is broader than the brain, bigger than the body

As neither skull nor skin impede energy flow, the mind emerging from it is not limited to the skull-encased brain nor the skin-encased body. Mind is fully embodied and fully relational.
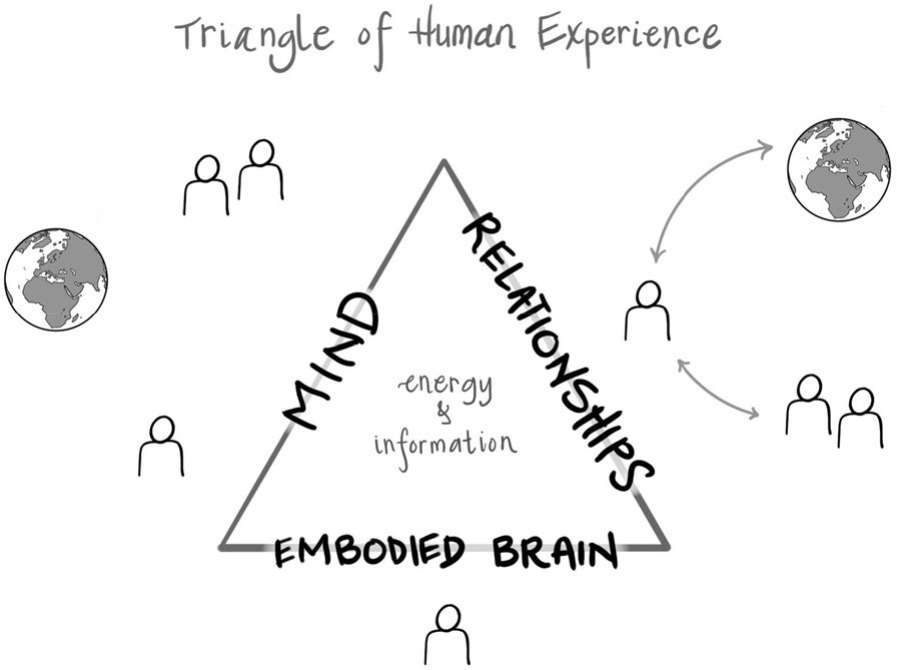


#### ENERGY is the movement from POSSIBILTY to ACTUALITY

One way of viewing the nature of energy itself is that it involves an emergence from potentiality into actuality. This can be envisioned as a “sea of potential” or “quantum vacuum” being the “formless source of all form” from which actualities arise, traversing a probability range from wide open with maximal uncertainty up to increasing probabilities and then maximal certainty as actualities. These can be named as a “Plane of Possibility” for the pool of potentialities, the source of all form; and then the increased states of probability can be labeled as “Plateaus” and the actualizations as “Peaks” [26]:
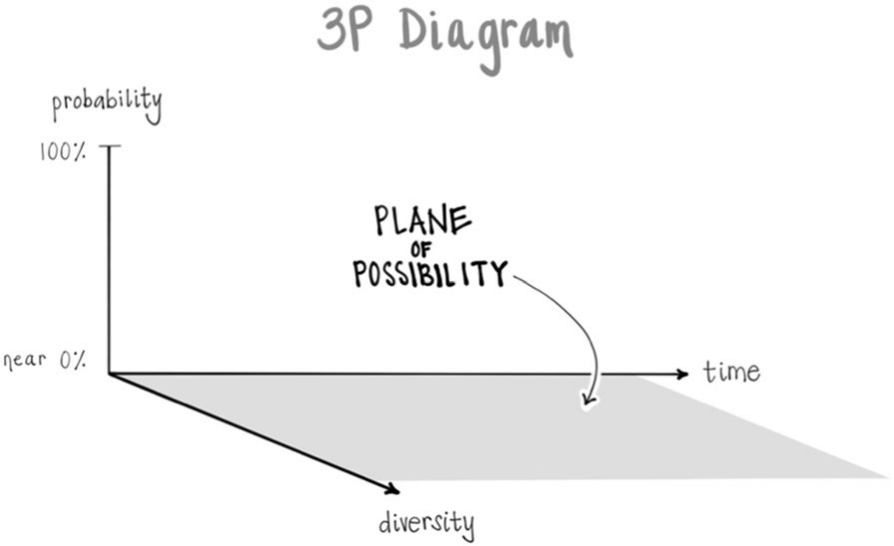



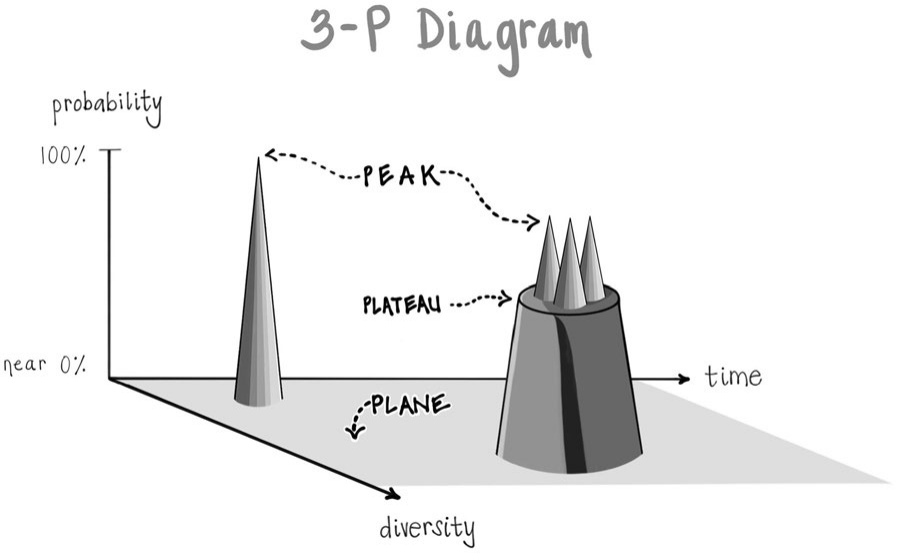


#### Energy flows as conduit or constructor of information

Energy flow can be direct as water through a hose in “conduition” and it can be formed into symbolic meaning as we construct energy into “information:”
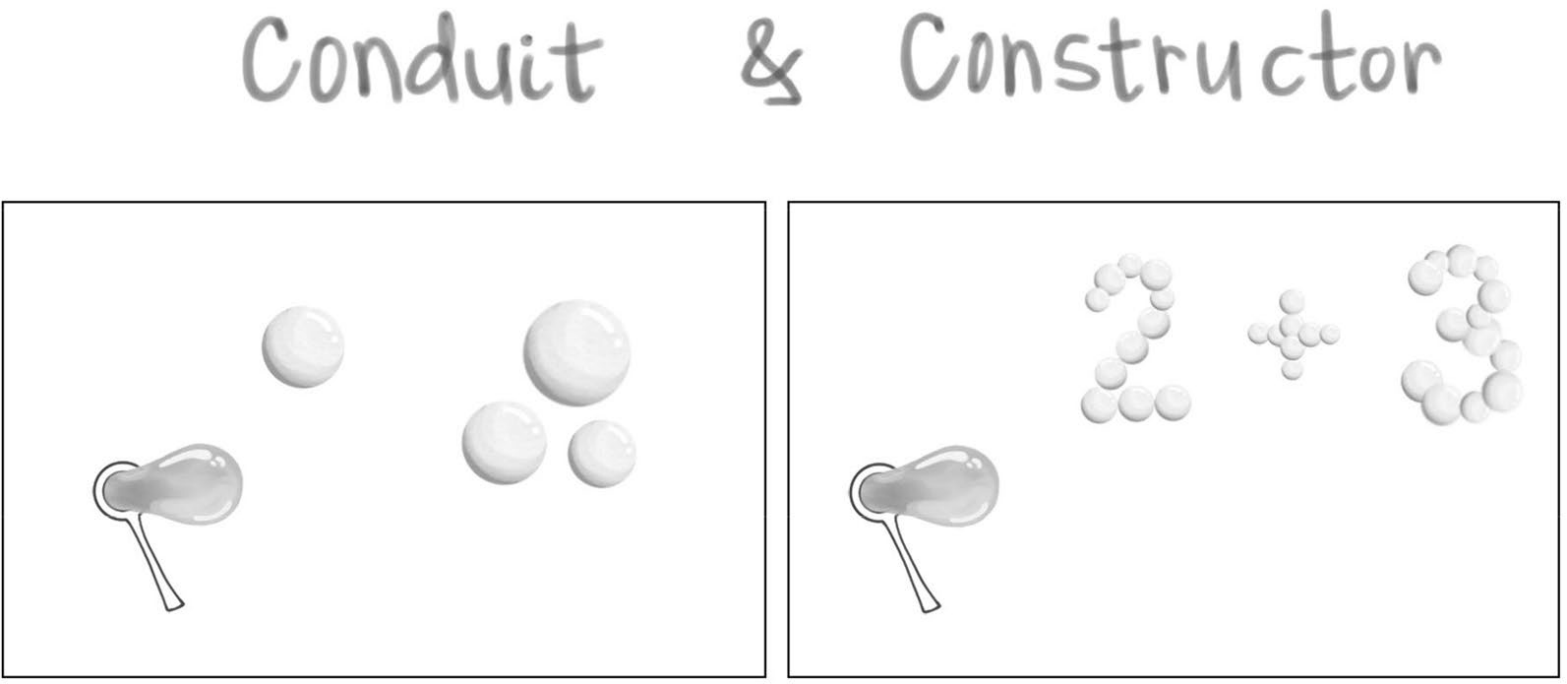


#### Our mind constructs CATEGORIES, CONCEPTS, and SYMBOLS

Information processing has many layers, and three of the core aspects of this cognition are the symbols we share with one another, such as linguistic symbols, and the concepts and categories these terms represent in our inner mental lives and our relational mental lives.

#### AWARENESS arises from a PLANE OF POSSIBILITY

After surveying tens of thousands of individuals and their experience of being in the “hub” of the Wheel of Awareness, it became apparent that if mind is an emergent property of energy flow, and if consciousness is an aspect of mind that includes the knowing of being aware and that which we are aware of, then awareness might correlate with the probability position of energy’s flow being in the Plane of Possibility:
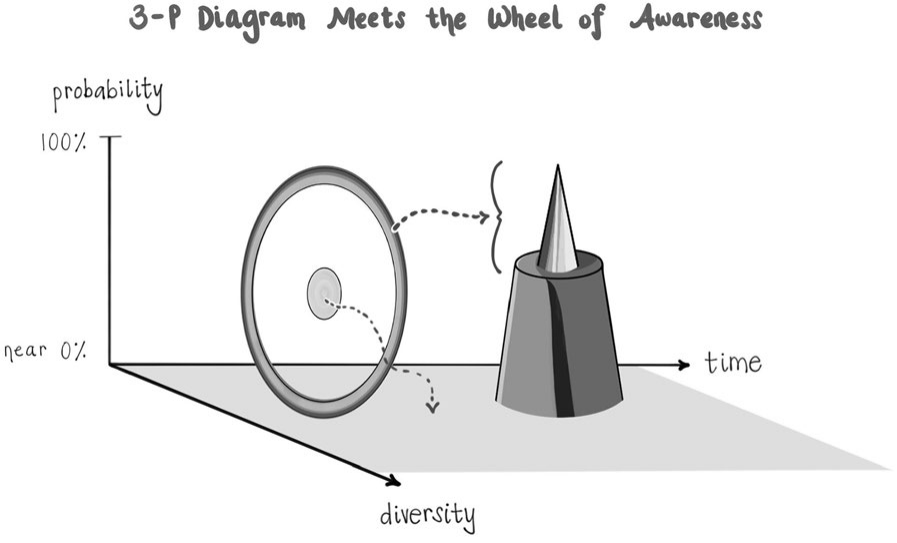


#### Plateaus and peaks may shape our “SENSE of SELF” and directly influence IDENTITY and BELONGING

The Plane correlates with awareness; Plateaus correlate with states of mind, mood, and intention; and Peaks correlate with specific thoughts, emotions, and memories:
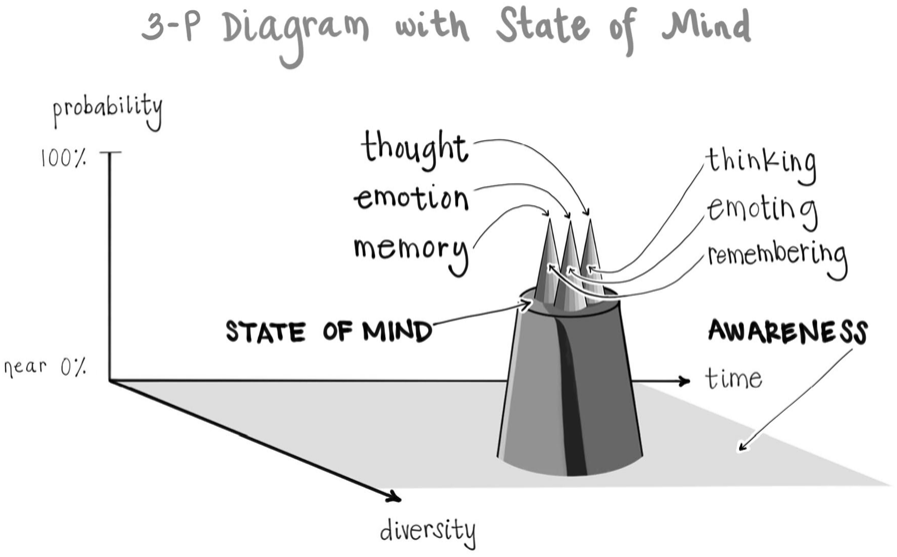


#### “SELF” can be viewed as a center of SPA: subjectivity, perspective and agency

If the constructed information term, self, signifies a center of experience, and if experience means energy and information flow, then that center of energy flow can have various locations and include three fundamental aspects of self-experience:

Subjectivity—the felt sense of being alive;

Perspective—the point of view from that center of experience;

Agency—the center of action.

#### Relational communication and evolution shape our construction in-group/out-group membership and the experience of a SOLO-SELF

Human beings have a long evolutionary history of dividing the world into “in-group” and “out-group” status from back at least fifty million years in our evolution as primates. Our contemporary experiences in families, schools, and the larger society shape our development of what is “self” and what is not. In individualistic societies, this cultural message of a separate self can be called a “solo-self” that views self-experience as centered only in the body.

#### Large groupings of people are vulnerable to in-group domination creating social injustice and caste systems & racism; misinformation; loneliness and addiction; and environmental destruction

A consilient perspective in science is that the disconnection inherent with the solo-self construction of modern culture may be the heart of the hidden symbol/concept/category of separation at the root of many of the major challenges to well-being for humanity, and for the natural world, at this time of the Anthropocene Era.

#### The wisdom of indigenous and contemplative teachings synergize with new contributions in the science of self, identity, and belonging

Though “modern culture” around the globe may teach that the Self is a separate, noun-like entity, Indigenous and contemplative teachings from around the world have independently offered a different view—of a self that is a more verb-like emergence embedded in all of humanity and in all of nature. When more recent views of “self” are synthesized from science in the last century and a consilience found with Indigenous and contemplative teachings from the last two millennia, a simple but serious and pressing perspective emerges that modern culture may be inculcating a “mistaken” identity of the self as separate, the solo-self, that may be leading to mental and medical illness, societal suffering, and environmental degradation of unprecedented intensity and speed. While this mistaken identity may be a result of the human mind and make us, humans, feel guilty or driven to ignore these findings, the positive and empowering news is that we can then use the human mind to shift that modern view of identity and lead to deep and long-lasting positive changes in our personal, public, and planetary health.

#### IntraConnection as an integrated self would weave the noun and verb ways of being, and the inner and inter self: me plus we equals MWe

In linguistic terms, in English, we don’t have a term to refer to the concept and category of the connectedness within the whole. *Inter*connection is a word indicating how the parts of a system connect with one another. But what term do we have to the subjective sense, the perspective, and the agency of the connections within the whole of the system? The new term, “*intra*connected”, offers one such linguistic symbol to embrace this notion that we can sense into the whole, take the point of view of the whole, and then act on behalf of the greater good of the whole. Rather than getting rid of a self, this view suggests that we have an *inner self* as me, an *inter* relational self as we, and the integrated self of *intra*connection that is Me plus We as MWe.

The subjective sense emerging from the Wheel of Awareness as a systemic way of exploring the mind’s consciousness gave rise to the notion that who we are is both noun-like entities of separation across what we name as “time” and “space” as well as verb-like emergent processes that are massively connected. In this verb-like unfolding, the science of energy reveals that we have two realms—one of the macrostates that Sir Isaac Newton articulated over three hundred and fifty years ago, and the other of microstates that the field of quantum physics has revealed in the last one hundred years. In this microstate realm of small things, of “quanta” or probability fields, there are no noun-like entities, only emergent processes. And the variable of time is not needed in quantum equations—there is no “arrow of time” as there is in the macrostate realm of entities obeying the Second Law of Thermodynamics in which we measure “time” as things moving toward entropy.

The proposal that awareness may arise when energy is in the “probability position” of the Plane of Possibility fits with this view of that Plane being the equivalent of the quantum vacuum. In addition to the description of the timeless quality of the hub, the term “empty-but-full” that is so frequently reported may correlate with this Plane being the “formless source of all form”—it is empty of form, but full of formlessness.

Though as clinicians and as scientists we are rarely invited to speak of the term love, the finding that that term is so often cited as the experience in the hub of the Wheel of Awareness raises a question that seems non-scientific but needs to be addressed: Why is love described in pure awareness? Consistent with Indigenous and contemplative teachings, and also described in many poets’ reflection on the nature of life, it seems that love may be a thread in the tapestry of life, perhaps of reality itself from which life emerges. In these repeated reports from individuals from around the world, that hub of the Wheel seems to have the three characteristics in the acronym, COAL: Connected, Open Awareness, Love.

In these ways, Interpersonal Neurobiology began as an effort to explore the nature of mind and mental health, and has expanded into a consilient effort to bring all disciplined ways of exploring the nature of reality into connection, collaboration, and conversation. In conventions on science and spirituality, one view of the latter term is that of a life of meaning beyond everyday survival and connection beyond the boundaries of the individual body. When we sense this spiritual view arising from our systematic exploration of the subjective experience of those integrating consciousness with a three-pillar practice like the Wheel of Awareness, we can see how the immersion in the hub reveals another “layer” beneath the surface in which time melts away, our connections with one another become felt, and love fills our conscious experience.

As Isaac Newton himself stated, “To myself I am only a child playing on the beach, while vast oceans of truth lie undiscovered before me.” (Venice Oceanarium quotation on the Venice Pier). Newton knew he could calculate the “location of celestial objects, but not the madness of men.” (Quotation from the birthplace of Isaac Newton, Woolsthorpe Manor, Lincolnshire, United Kingdom). Might it be that what physicists have now determined—that we live in one reality that has at least two realms with their own distinct properties of the macrostate Newtonian classical physics of noun-like entities bounded by time and space separation, and that of the microstate quantum realm of verb-like processes whose connections are not impeded by Newtonian notions of time and space—might in fact relate to what is true beneath the surface of that ocean? William James, the grandfather of modern psychology, stated a similar sentiment: “We are like islands in the sea, separate on the surface but connected on the deep.” (Venice Oceanarium). Just as the Pando Populus Forest in Utah reveals, forty-eight thousand seemingly separate Aspen trees are really outer trunks linked in one massive root ball just inches beneath the surface. Could the modern human mind be constructing a shared informational symbol of “self” that we equate unquestionably as the individual? Might the limitations of our field of mental health be that we’ve taken in this error without assessing its validity? To take in the subjectivity, science, and spirituality and then truly be of service for reducing the suffering in the world, perhaps this view of the solo-self is like a splinter in the psyche of modern society. How might we remove that splinter and live in a more intraconnected, integrated way as a family of human beings and as a family of all living beings on Earth?

### If mind is embodied and relational, what and where is the self?

One of the take home messages from all of this work for clinical and cultural implications and applications is that the human mind is as much relational as it is embodied. And this view also suggests that when we see mind at a minimum as including our subjective sensation of being alive, our subjectivity, then the mind is not just up in the head. Yet in the field of medicine for 2500 years since the time of Hippocrates [12], it is a common view that “mind is what brain does.” This perspective, combined with the individualism of our modern cultural view as discussed above, places the mind, and the sense of self it constructs, into the body of the individual. We’d like to humbly suggest that in our field of mental health, by viewing the self as equivalent to the individual, just as we’ve often viewed the mind as a synonym for brain activity alone, we’ve fallen for a vulnerability we have as a species toward a mistaken identity that is not serving us well as a human family on Earth, nor as a profession of mental health practitioners. It is important to understand why we’ve had these views, and here’s one possibility for how to understand what may have happened.

The world and our lives in it are filled with uncertainty. The human brain can be considered an anticipation machine with its innate drive to detect patterns and predict outcomes to prepare for what is happening next to increase our chances of survival. When that same anticipatory brain uses language to describe the world, the *nouns* it forms in linguistic representations reinforce a concept that there are *entities* in life, things one can hold, for example, that have defining features. In contrast, language can also symbolize the concept of the *processes* that are happenings in the world with *verbs*. With verbs we sense the unfolding of experience that is often filled with uncertainty—it lacks the predictability of the noun-like sense of certainty that an entity has with a certainty in its features. If we can construct an identity as an entity we achieve an illusion of certainty. Information construction as a narrative self, the aspects of our identity that tell the story of “who we are” to ourselves and other selves, may come to believe what culture has been telling it, what parents may have been saying, what school has told it: that self is a noun-like entity. This view of a separate self lends a sense of certainty to our minds, but it may be not only a limited part of a larger story, it actually might also be a lethal lie. A lie in that it is not telling the truth; lethal in that it may have life-and-death consequences for individuals, for our human family, and for the future of life on Earth.

So, what this brings up is that life can be perceived as nouns and as verbs. And in modern culture, we start to think of the self as a noun, as a fixed entity. And yet there’s a whole other realm where we can experience life as a verb-like unfolding. This may correlate with the physics’ view of our living in two realms of one reality, a noun-filled macrostate realm and a verb-filled microstate realm. And this distinction in linguistics is not just a matter of words, as symbols are simply the tip of an iceberg of information processing. Beneath the symbol of a word are concepts and categories, as we’ve seen, and these are like Plateaus in our probability diagram filtering what we ultimately experience as thoughts, or emotions, or memories. What those categories reveal is a way of thinking that can be called *linear thinking* in contrast to *systems thinking*. Returning to physics from 350 years ago, Isaac Newton’s view of what is now called “classical” physics looked at entities as nouns—such as an apple falling from a tree or planets whirling through space. Even in the birthplace of Sir Isaac, that quote from the scientist says that though he could predict the location of celestial objects with his calculations, he could “not predict the madness of men.” Perhaps we in the field of mental health should take his reflections to heart and realize there are other properties to the mind than what we see with our Newtonian vision of the macrostate world of matter. Since Newton’s time, we also now have a century old quantum physics view that, because of technological advances, enables us to observe and consider the unfolding of quanta, the fundamental units of energy. Quanta are probability fields, and their properties are more akin to massively connected verbs—interwoven processes emerging without the clear boundaries of entities, without being nouns, without being “things” separated in Newtonian dimensions of time and of space. It may be that our minds have the ability to adjust an “identity lens” that allows us to move back-and-forth between these two realms, feeling the reality that we do live in a body, and then opening to the experience of COAL in pure awareness as we sense the connection, open awareness, and love at the heart of our direct subjective experience.

If the proposal that mind is an emergent property of energy flow is accurate, then we’ve seen that this flow can be both inside the individual—the “me”—and it can be between the individual and the world of other people and the physical environment—the “we.” If mind is an emergent property of energy, it is *both* embodied and relational. And if this proposal is true, then it also means that turning to the science of energy may yield important insights into the nature of mind itself. For example, if entities are the macrostates that Newton was able to see with his eyes and whose actions could be predicted with his classical physics equations, then this level of reality is supported by a robust empirical and practical set of experiences. And at the same time, our one reality has the microstate realm in which quanta such as electrons and photons can be carefully studied and their properties of probability described that are quite distinct from those of the macrostate, classical, Newtonian realm of our one reality. Just as we have the properties of walking on land as distinct from those of swimming in water, we have distinct properties in the macrostate and microstate realms. If mind is an emergent property of energy, if that is indeed true or even partially true, then one would predict that the study of energy itself might reveal ways our mind functions.

One possible explanation for the commonly stated feeling of the hub being “empty but full” is that the energy position of this open state of awareness might be in what physicists have named as the quantum vacuum or sea of potential: the “formless source of all form” as quantum physics professor emeritus Arthur Zajonc calls it, that is the “ground of being” of our experience of energy. If, in the field of mental health, we take seriously the proposal from physics that energy is “the movement from possibility to actuality,” we are then equipped as a field to see how working with the mind involves empowering our clients to move from this open space of maximal uncertainty upward toward various degrees of probability and then certainty as actuality.

What this survey of the experience of those doing the Wheel of Awareness also suggests is that mental training might be able to reveal how the experience of being aware arises from an energy state that was wide open—one that felt “empty but full” and involved a sense of wide open connections “to everyone and everything”—that is sometimes attributed to mystical states of awe. These awe states [13] are directly correlated with enhanced well-being and a widening of the sense of self. As professor Keltner and I surveyed workshop participants doing the Wheel of Awareness, we found the degree of mystical sensations such as awe were similar to what they might experience in the research studies of psychedelic interventions. And interestingly, the same conditions that were independently found clinically to help with mild to moderate depression, anxiety and trauma, and terror in the face of death, were also what the modern studies of psychedelic use are pointing to as areas of clinical efficacy [3]. In the entropic brain hypothesis of Robin Carhart-Harris, what is found is highly uncommitted—uncertain—brain states during effective psychedelic treatment protocols [4, 5]. Could this highly uncertain state be equated with the hub of the Wheel? Might the efficacy of awe, psychedelic treatments, and perhaps the Wheel in future studies, be due to moving the energy position beneath pre-existing Plateaus and their repeating Peaks toward the source of other options—the Plane of Possibility?

How might doing a practice like the Wheel of Awareness that trained the individual to access the “hub of the wheel” that might correlate with this Plane of Possibility be helpful? Why would such three-pillar mind practices promote well-being in the medical and neurological ways we described earlier? We can propose that seeing the mind as an emergent property of energy flow helps us to answer these questions.

This statement offers an initial step to address how mind can change the health of the body and the relational connections that influence our well-being: where attention goes, neural firing flows and neural connection grows. With mental training, a state created in the practice leads to neuroplastic changes that alter the structure and function of the brain (See Davidson and Goleman *Altered Traits*). The way one uses the mind can change the brain in four ways: Change the synapses called *synaptogenesis* that connect neurons in certain areas; Grow new neurons with *neurogenesis*; Lay down an important sheath, *myelin*, that makes the neurons connect with each other more effectively and time their firing in a more coordinated way; and, Change the molecules that alter how we respond to experience—our epigenetic *regulation*.

When more integration is cultivated in the brain, nine functions that are associated with an integrative aspect of a particular region, the prefrontal cortex, are enhanced: (1) the ability to regulate bodily state; (2) Attuned communication, which means focus your attention on the inner experience of another human being and even in reflection with yourself; (3) To balance emotions, to be fully aware of them and embrace them; (4) To modulate fear; (5) To learn to respond flexibly to a stimulus that comes to you and not just be automatic: to pause before automatically reacting; (6) Insight, to have what’s called autonoetic consciousness, a self-knowing awareness which is what insight is, connecting the past, present and future; (7) Empathy, to feel another person's feelings, morality and intuition; (8) Compassion—to feel another’s suffering, reflect on what actions to take, and then to take those actions on behalf of reducing suffering; and (9) Morality—to act on behalf of the greater good and to honor integration at the heart of moral living.

These nine correlates of neural integration are also the heart of what is sometimes called, “being mindful.” And in many ways, these are the outcomes of secure attachment between a child and a parent. Integration within emerges from integration between.

Returning to where we began, the psychache as the common experience of those with suicidal thoughts and actions, let’s see if we can synthesize these many integrative notions of Interpersonal Neurobiology into a reflection here as we come to bring this discussion to a close. When a person is in a state of mind that’s suicidal and they can’t think of any other choices but those particular peaks arising, our job is to give them a connection that allows them to drop beneath that Plateau to the Plane of Possibility. This may begin with our own inner practice where we can sense that there is a generator of diversity, that Plane of Possibility or sea of potential, that formless source of all form, that rests beneath those limiting Plateaus and their suicidal peaks.
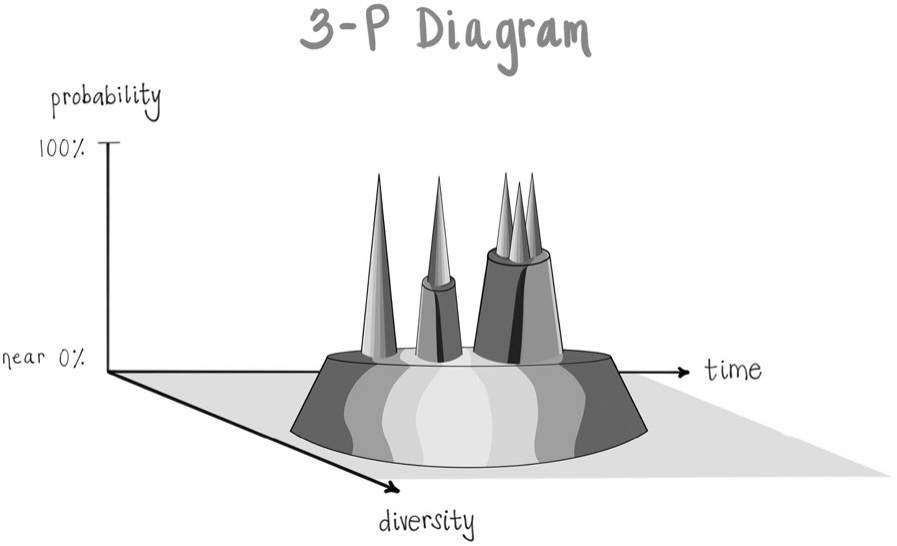


As a clinician, sensing this Plane of Possibility is the hub of the Wheel of Awareness. You are working with the mind, which is fully embodied and fully relational. Understanding the mind as an emergent property of energy, you now have a visual image, for yourself, and sometimes even showing this to clients or patients is helpful, of where, at the present moment, the mind is taking you or the other person. You are working also with the self-experience of the patient, and knowing the acronym SPA, you can get a feeling for the S of sensation and subjectivity, the P of perspective and point of view, and the A of agency and action in the world. Where is the individual at this present moment? How is their identity lens in its flexibility? Is it stuck close up so that the self is an isolated solo-self experience? Are they imprisoned by a low-lying plateau of isolation only allowing certain senses of self and thoughts and emotions of being alone to arise? Or, do they have experience feeling a more expanded sense of identity, a wider sense of who they are in their connections with other individuals or nature? Can they access that place of connection which is filled with the realization that though this Plane of Possibility is maximal uncertainty, it is really the source of other options—of freedom and possibility. In that more expanded identity lens view arising from this more open awareness, can they broaden their belonging beyond their individual body?

When people have psychache and are thinking of killing themselves, their SPA of self is filled with suffering as chaos and rigidity: their Sensation is rigidly full of pain, their Perspective is incredibly restricted and they don’t see any future hope, and their Agency feels unable to do anything and filled with a chaotic sense of despair and lack of efficacy, they feel helpless and hopeless. So in deep ways, a suicidal self includes these three aspects of self that are not integrative, they are filled with the chaos and rigidity of psychache.

Now what's remarkable is that when you look at the role of that Plane of Possibility, you can see why and how consciousness that emerges from that Plane allows you to work with someone who's in a suicidal state, work with someone who's been in trauma, to actually have the courage to drop into that Plane of Possibility. If you’ve experienced this yourself, doing the Wheel of Awareness or another practice that allows you regular access to that COAL state of being connected with open awareness and love, you come prepared to literally be present and be your PART—with presence, attunement, resonance, and trust. It is this PART we play that enables our clients and patients to “feel felt” and to sense directly the widening of their identity, a broadening of their belonging.

As mental health clinicians, we can work at that level of the Plane of Possibility, and various domains of integration in the therapeutic journey can emerge from it. What seems apparent is that the Plane is a form of portal through which integration is freed to emerge as the natural push of a complex system to optimize its self-organization. In energy terms, we acknowledge within consciousness the differentiation of the Plane from the mind’s Plateaus and their limited Peaks.

### Regulation arises from integration; integration is the basis of well-being

Optimal regulation—both internally and relationally—arises from integration. When we honor differences and promote compassionate linkages, interpersonal relationships thrive. And when we look to the neural networks involved in all forms of regulation, including the regulation of attention, emotion, thought, memory, self-awareness, and morality, these are dependent upon the regions of the brain that link differentiated areas to one another. In this way, when we consider that mental dysfunction emerges with difficulties in regulation, we can understand how mental suffering—experienced in the broad dysregulated states of chaos and rigidity—is an outcome of impaired integration. Attachment relationships that are secure early in life promote optimal inner regulation by way of cultivating the growth of integration in the brain. An integrative brain, in turn, enables optimal interpersonal regulation to develop, just as these secure relationships had their impact by way of integrative communication, the honoring of differences, for example, between parent and child, and the cultivation of emotional linkages that then stimulate the growth of further neural integration in the child. In turn, the child with the resilience of an integrated inner system can then go out into the world and create and sustain mutually rewarding, health-promoting, close interpersonal relationships. Neural and relational integration mutually support one another.

If integration is the natural outcome of a complex system’s emergent property of optimal self-organization, then why would there ever be a compromise in this innate drive toward harmony and health? We can propose that certain experiential factors, such as trauma or sub-optimal attachment relationships, set the relational world of a child to block that natural drive of the brain to become integrative. Developmental trauma, for example, has impediments in the growth of such integrative regions and their functions as the hippocampus, the corpus callosum, the prefrontal cortex, and the connectivity of the connectome. At the other end of etiology, certain psychiatric conditions such as bipolar disorder and autism spectrum also have blockages to integration: Bipolar disorder with impaired linkage between the prefrontal cortex and sub-cortical amygdala regions and autism with impaired differentiation of the cortical regions [6, 9, 16]. In this way, both experiential and non-experiential primary causes of mental suffering can share the common finding of blocked integration as the basis of mental challenges.

When we offer a definition of one facet of mind as an emergent, self-organizing, embodied and relational process that regulates the flow of energy and information, we are seeing that the mind’s regulatory function would entail at least two features of regulation: monitoring and modifying. Sometimes individuals have difficulties with one or both of these essential aspects of regulation and they cannot effectively sense and shape, track and transform, the inner and inter energy flow in their lives. A clinician, then, can sense the chaos and rigidity that an individual, couple, or family may be experiencing and teach the capacity for monitoring and then modifying energy flow so that the innate drive toward integration can be released. In this way, psychotherapy works with the natural push toward health that may have been blocked for innate or experiential reasons. Often these two features of inborn proclivities and adverse experience work together to create vulnerability in the form of compromises to integration [1]. These compromises may be seen in one or several of many “domains” of integration.

Nine domains of integration may be important throughout the lifespan. *Integration of consciousness* enables the Plane to be differentiated from Plateaus and Peaks, and in the metaphor of the Wheel practice, this means distinguishing Hub from Rim of the wheel. Within the brain, *bilateral integration* of the two sides of the brain involves linking the differentiated right side’s broader focus of attention, non-verbal communication, and integrated map of the interior of the body with the left side’s mode of more narrowly focused attention, linguistic processing of information, and logical methods of reasoning. *Vertical integration* allows people to get more in touch with the sensation that's in their body in what in science we call, “interoception” or the sixth sense, in which we feel the interior sensations of muscles, bones, and organs. *Memory integration* involves the differentiation of past, present, and future in what's called implicit and explicit memory processing. *Narrative integration* is how we make sense and find meaning in life so that someone who's suicidal can find a deep, truer narrative than the lie of the separate self.

Narrative is not just the stories we tell, but how we come to live our lives. If culture—embedded in our society’s messages, our teachers’ lessons, our parents’ beliefs—tells us that who we are is a separate solo-self, we come to believe this to be “the story of our lives.” Unquestioned, it is simply seen as the truth, just “the way things are.” In modern culture, though this is a narrative that is false, it is naturally what we try to live into and rarely question as being valid.

With *state integration*, we can differentiate layers of self-experience that involve our individual bodies, our connection with other people, and our being a fundamental part of nature. The lives we lead in modern culture may see the self-state of separation to be our defining feature, rather than the states of being expansive with a sense of wholeness. The classically named “self-transcendent” emotions of awe, compassion, and gratitude may be more usefully referred to as “self-expanding” emotions and are thought to deeply aide in the creation of well-being [2, 13]. Living with a sense of self and mind that is constricted and believing something that's false may involve a sense of disconnection in modern times that may be core part of the etiology of the increasing levels of suicide, loneliness, addiction, anxiety, and depression rampant in modern culture, especially in the United States. *Temporal integration* is a form of linking differentiated aspects of our lives that has to do with our experience of time and the way we wrestle with the fact that we know we're going to die. What do we do with that existential issue and our underlying longing for certainty? Our probability diagram helps us envision how embracing the reality of uncertainty is a pathway toward mental health and resilience. Part of this involves embracing apparent paradoxes of longing for immortality in the face of the reality of mortality, longing for permanence in the face of impermanence, longing for certainty in the face of uncertainty. Our ninth domain is that of *identity integration*, with our sensation, perspective, and agency of identity that is not just inside the body as an individualistic self-construction; and this integration is not about going to the other extreme of losing any individuality and being only relational. With identity integration we can learn to adjust an identity lens as we realize the reality of our inner me, our inter we, and our intraconnected identity as MWe.

In medicine when we encounter a cell that acts as if it is separate from the rest of the cells of the body, we call it cancer. It may well be that viewing self as identical to the individual has inadvertently created a cancer of modern culture and we are living with the chaos and rigidity of the suffering that arises from such a non-integrative mental construction. We are inviting you to consider that the psychache of suicide and the suffering of modern culture are each stemming from the same issue of impairments to integration. The lie of the separate self, the solo-self only defined by the individual's skin encased body, is a lie that is creating the conditions of disconnection underlying suicide for individuals and for our modern world. The solo-self construction of identity creates massive mental suffering for the individual. It differentiates human beings from each other, setting the stage for racism and social injustice. And the solo-self view of who we are differentiates the human species from other species so that we treat Earth like a trash can.

We propose that we in the field of mental health, for those of us who know how to work with the mind and specifically with the psychache of suicide, for those who know how to think deeply about well-being, we need to apply our knowledge and skill as we look deeply into our modern culture. We in this magnificent field are at a crucial time in the evolution of not only humanity, but of life itself on this precious, fragile common home, this planet we’ve named, Earth.

We do live with a me, an aspect of a multidimensional self that lives inside the individual. This is an inner self, the SPA of the body’s sensation, perspective, and agency. Me is real and it is an important differentiated aspect of our fuller identity. And we also have a we that is our relationships with people and the planet. This is our inter self, an emergent aspect we call our relational mind and its experience of self as this sensation, perspective, and agency of the relational connections we have.

You might ask, “How can who I am—my subjective sensation of being alive, my perspective, and my agency for being an initiator of action—be both a me and a we?” We want to suggest to you that we have the opportunity to integrate these two fundamental sources of self-experience—of an inner Me and an inter We. To honor the differences between the inner and the inter and link them without losing their integrity, their differentiated nature, their essence. One way to do that is to look at what we’ve called *intra*, to look at the whole system and how that whole system is intraconnected. It is possible to sense into, perceive from, and act on behalf of this larger intraconnected whole. We can name this in our constructive, communicating minds as Me plus We is MWe, an intraconnected integration of the inner and the inter, where the differentiated aspects that are linked in an integrated flow do not lose their integrity in the integration. Our hope is that in our exploration together here, as people who are so devoted to the well-being of humanity and all life on Earth, that we can actually feel the ache of the psyche of the individuals and the ache of the psyche of humanity and the ache of the living systems on Earth, and reimagine what mind and self truly are to support us in finding the deeper meaning in our intraconnected nature.

Thank you for being on this collective journey to understand and bring health to the mind.

## Data Availability

Not applicable.

## Footnotes

1 This article is based upon a presentation for a Lifetime Achievement Award to the first author from Sapienza University at the International Symposium on Suicidology and Public Health in 2022. Chloe Drulis has worked closely with the ideas and applications of Interpersonal Neurobiology, and it is an honor to weave this article with her to document this interdisciplinary framework for understanding the mind and mental health.
